# No Modulation of Visual Cortex Excitability by Transcranial Direct Current Stimulation

**DOI:** 10.1371/journal.pone.0167697

**Published:** 2016-12-09

**Authors:** Sabrina Brückner, Thomas Kammer

**Affiliations:** Section for Neurostimulation, Department of Psychiatry, University of Ulm, Ulm, Germany; University Medical Center Goettingen, GERMANY

## Abstract

Measuring phosphene thresholds (PTs) is often used to investigate changes in the excitability of the human visual cortex through different brain stimulation methods like repetitive transcranial magnetic stimulation (rTMS) or transcranial direct current stimulation (tDCS). In several studies, PT increase or decrease has been shown after rTMS or tDCS application. Recently, using PT measurements we showed that the state of the neurons in the visual cortex after rTMS might have an influence on the modulatory effects of stimulation. In the present study we aimed to investigate whether visual cortex activity following stimulation influences the modulatory effects of tDCS as well. In a between-group design, anodal or cathodal tDCS was applied to the visual cortex twice per subject, with either high or low visual demand following stimulation. We observed no modulation of PT neither directly following both anodal and cathodal tDCS nor following the visual demand periods. We rather found high inter-individual variability in the response to tDCS, and intra-individual reliability in the direction of modulation was observed for cathodal tDCS only. Thus, our results do not confirm the modulatory effects of tDCS on visual cortex excitability published previously. Moreover, they support the confirmation that tDCS effects have little reliability on varied TMS outcome measurements.

## Introduction

An essential method for exploring the modulatory effects of different brain stimulation techniques is measuring cortical excitability by single-pulse transcranial magnetic stimulation (TMS). In the motor system, this is often realized by comparing the amplitude of motor evoked potentials (MEPs) before and after repetitive TMS (rTMS) or transcranial direct current stimulation (tDCS). Whereas low-frequency rTMS decreases MEP amplitudes (e.g., [1, 2], high-frequency rTMS increases it (e.g. [3, 4]). For theta burst stimulation (TBS), continuous application (cTBS) decreases and intermittent application (iTBS) increases MEP amplitudes [5]. Applying tDCS, a decrease of MEP amplitude was observed with cathodal and an increase with anodal polarity [6]. However, there is evidence for interindividual differences in the response to all of those methods in the motor system, i.e. rTMS [7, 8], TBS [9] and tDCS [10]. In case of the visual system, beyond visual evoked potentials (VEPs) usually PT is used to investigate changes in visual cortex excitability. Phosphenes are commonly defined as elementary visual percepts which can be evoked by single-pulse TMS to the visual cortex [11]. Various studies showed that the modulatory effects known from the motor system can be observed in the visual system as well: increased PTs were found after low-frequency rTMS [12–14], cTBS [15] and cathodal tDCS [16, 17]. Following high-frequency rTMS [14] or anodal tDCS [16, 17] a decrease of PT was observed. Interestingly, some studies failed to observe the expected modulatory effects of different brain stimulation methods on visual cortex excitability. For instance, “facilitatory” 5Hz rTMS produced an inhibitory effect [18], iTBS had no effect on PT [15] and anodal tDCS failed to produce any after-effect on contrast sensitivity [19]. Recently, it was suggested that at least TBS effects on visual cortex excitability depend on the state of the neurons even after TBS [20]. For tDCS, it was shown that neuronal activity before or during stimulation [21, 22] can modify the direction of modulatory effects. High visual demand caused by reading during tDCS decreased PT independent from polarity of stimulation [23]. The role of enhanced visual cortex activity directly after tDCS has not been explored yet. Additionally, in a systematic review it was shown that there is little-to-no reliability of neurophysiological effects beyond MEP amplitude in tDCS studies [24]. However, in their analysis studies investigating PTs were not included. Therefore, our aim was to investigate the reliability of the effects of both anodal and cathodal tDCS on visual cortex excitability using PT measurements. Additionally, we aimed to examine whether the state of the neurons following tDCS, as varied by high or low visual demand after stimulation, has an influence on the expected modulatory effects. Thus, the design of the present study was exactly the same as published previously [20], but applying tDCS instead of TBS. Our hypotheses were: (a) anodal tDCS will decrease PTs and cathodal tDCS will increase PT [16, 17]; (b) if subjects are exposed to low visual demand following tDCS, the modulatory effect will last for at least 10minutes [16]; and (c) if subjects are exposed to high visual demand following tDCS, the modulatory effect will be modified, i.e., there will be a decrease in PT independent from tDCS polarity [23].

## Materials and Methods

### Subjects

Altogether, 47 subjects were recruited for the study. Subjects with metallic implants, prior history of psychiatric or neurological disorders, major medical illness, drug abuse or any medication with the exception of contraceptives were not included. Eight subjects gave no written informed consent, five showed instability in phosphene perception (no PT could be calculated from data), and due to methodological problems with our navigation system we lost data in two cases. Thus 32 subjects remained for the experiment. They were divided into two groups with 16 subjects each receiving either anodal (mean age 22.8 ± 3.7 years, 7 male) or cathodal (mean age 22.8 ± 3.0 years, 9 male) tDCS. As revealed by the Freiburg Visual Acuity Test (FRACT [25]), subjects had normal or corrected-to-normal visual acuity. The study followed the Declaration of Helsinki and was approved by the ethics committee of the University of Ulm. All subjects included gave their written informed consent and were paid for participation.

### Experimental design

**Fig 1:**
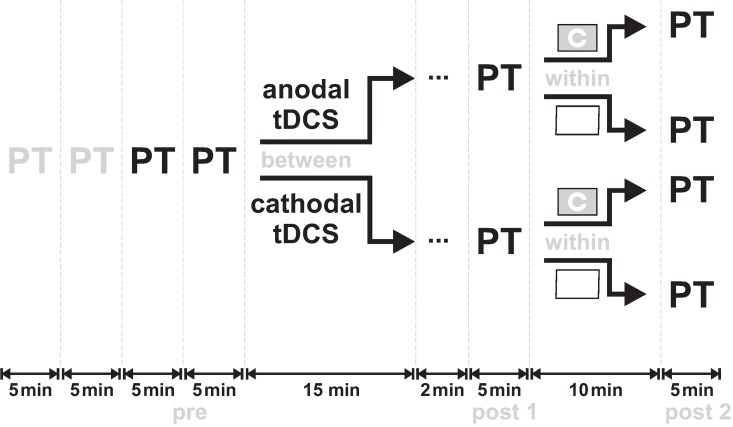
Design of the study. Phosphene threshold (PT) was measured four times at the beginning of each session, with the first two being discarded as practice runs and the other two averaged as baseline PT value. In a between-group design, either anodal or cathodal tDCS was applied to the visual cortex for 15min at 1mA. Two minutes after the end of tDCS, PT was established again (post 1), followed by a 10min period of either low or high visual demand. Finally, PT was measured again (post 2). The design as well as the scheme was adopted from our former study [20] and adjusted for tDCS.

The design of the experiment was almost identical to that used in our former study [20] and is illustrated in Fig 1. Sitting in a comfortable chair during the whole session, subjects received biphasic magnetic pulses delivered with a Magpro X100 stimulator (MagVenture, Farum, Denmark) and a figure-of-eight coil MC-B70 placed over the occipital cortex. For details regarding phosphene perception criteria, familiarization procedure and PT measurements, see our former study [20]. For each subject, the coil position inducing the strongest phosphene perception was determined (“hot-spot”, cf.[20]) and kept constant using the frameless stereotactic positioning system BrainView (V2, Fraunhofer IPA, Stuttgart, Germany, cf. [26]).

At the beginning of each session PT was measured four times, whereof the first two were discarded as practice runs and the other two were taken as baseline PTs (pre). Then, either anodal or cathodal tDCS was applied. Identical to our former study [20], two minutes after the end of stimulation PT was measured again (post 1), followed by a 10min period of either low or high visual demand in a within-subject design. Thus, subjects completed two sessions each, with at least 48h in between. In the session with low visual demand, they had to keep their eyes open, looking at a white wall. High visual demand was realized by a visual acuity task (details in [20]). In brief, in the center of a screen a Landolt C optotype was presented for 10ms with its gap oriented in one of four potential directions. Subjects had to indicate the direction of the gap by pressing one of four buttons. The size of the Landolt C optotype varied following a 2:1 staircase in steps of one pixel, and after seven reversals of the staircase the run was terminated and the next run was started to avoid fatigue. Since one run lasted 1.5-2min, subjects had to complete 5–6 runs within the 10min period of high visual demand. The task was used for induction of high visual demand only, the visual acuity thresholds were not analyzed further (c.f. [20]). Following the low or high visual demand period, PTs were measured again (post 2).

### tDCS

TDCS was delivered by a battery-driven direct current stimulator (NeuroConn GmbH, Ilmenau, Germany) through a pair of 7x5cm saline-soaked surface sponge electrodes. The concentration of the NaCl solution was 15mM [27]. For anodal stimulation, the anode was placed over the previously established phosphene hot spot and the cathode over CZ and vice versa for cathodal stimulation. TDCS was applied for 15min at 1mA with a ramping period of 15s. Recently, it was suggested that tDCS effects may depend on the timing of the task with respect to stimulation [28]. If the task is administered post-tDCS, as in the present work, subjects’ cognitive activity during stimulation must be carefully considered [28].For that reason, subjects performed an acoustic oddball task during tDCS. They listened to a sound file containing a sequence of two different beeps (300Hz and 500Hz, 0.2s each) in randomized order, and they had to count the rarer beep. Thus, cognitive activity was standardized between subjects. Furthermore, it enabled us to control for general attention, since a large discrepancy between the subject’s count and the correct value would have indicated if a subject felled asleep during stimulation.

### Data analysis

Data were first analyzed with regard to normal distribution using Shapiro-Wilk’s test. Pre and post PT values were subjected to repeated- measures analyses of variance (rmANOVAS, Statistica V.12, StatSoft GmbH, Hamburg, Germany) for each group separately. Sphericity requirements were assessed using Mauchley’s test. Post-hoc analyses were performed using Newman-Keuls test. Several correlation analyses were performed by calculating Spearman’s rank correlation coefficient.

## Results

### Baseline values

At the beginning of each session, baseline PT was measured four times. The first two measurements were discarded as practice runs, the other two were analyzed with regard to stability of baseline PT. Data were subjected to an omnibus rmANOVA with the between-factor GROUP (anodal/cathodal) and the within-factors SESSION (high/low visual demand) and MEASUREMENT (3/4). There was no main effect for any factor and no but one interaction (SESSION*GROUP). In Table 1 F- and p-values are reported. Post hoc Newman-Keuls test revealed that, in the anodal group, baseline PTs were significant lower in the session with low visual demand.

**Table 1:** Analysis of baseline stability.

effect	F _(1,30)_	*p*
GROUP	1.63	0.21
SESSION	3.69	0.06
MEASUREMENT	1.08	0.31
SESSION×GROUP	6.22	0.02
MEASUREMENT×GROUP	0.57	0.46
SESSION×MEASUREMENT	0.12	0.73
SESSION×MEASUREMENT×GROUP	0.16	0.69

**Fig 2:**
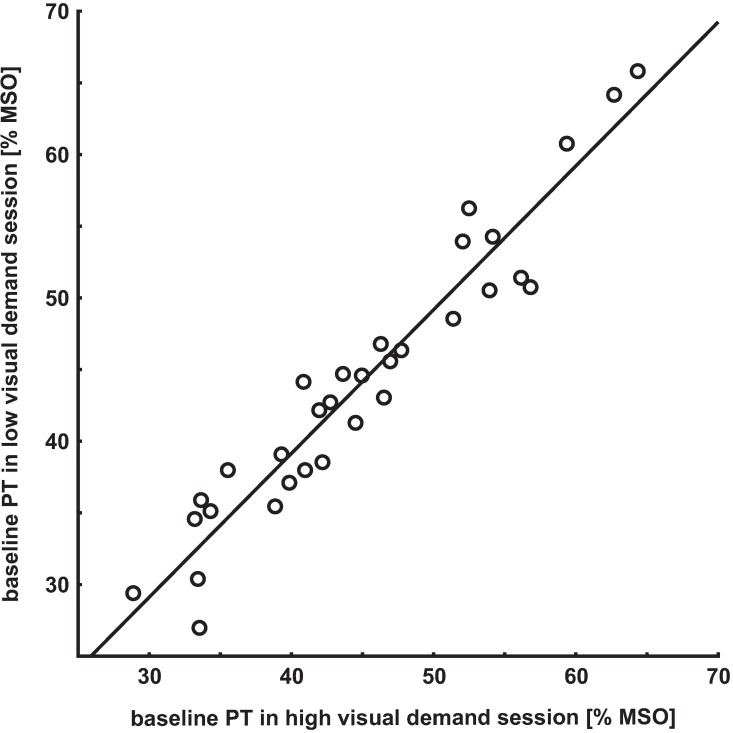
Correlation between the baseline PTs of the two sessions. Although baseline PTs differed significantly between sessions in the anodal group, a high correlation was observed (r_s_ = 0.96, p<0.001).

Since stability in baseline PT within sessions was observed, the two pre-values of each session and participant were averaged as pre-tDCS values. Although in the anodal group baseline PTs differed significantly between the two sessions, a high correlation (r_s_ = 0.96, p<0.001) between the baseline PTs of the two sessions was observed (see Fig 2).

### Anodal tDCS

**Fig 3:**
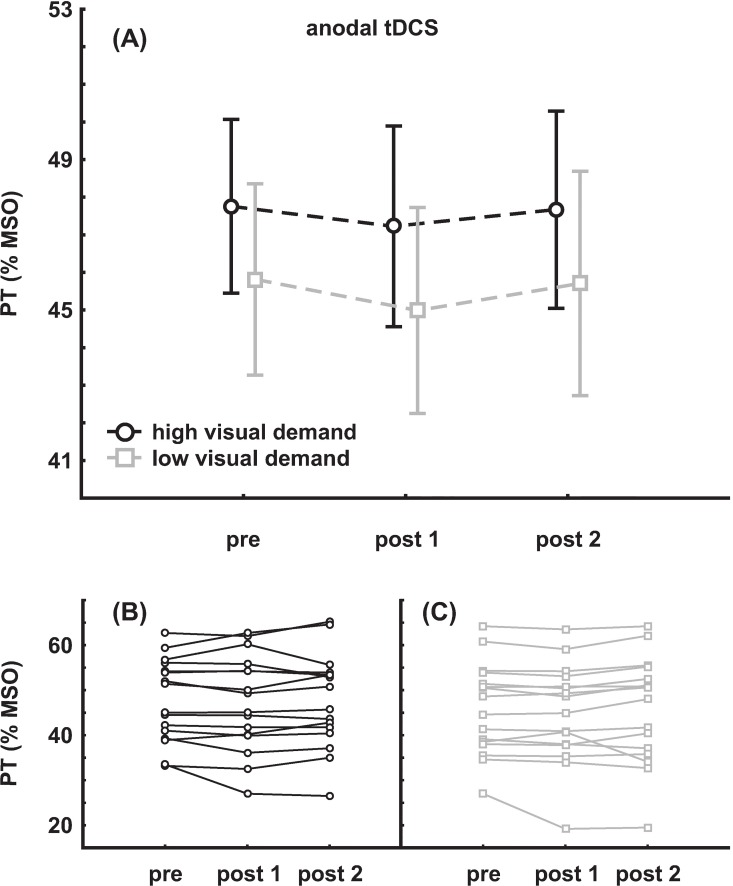
Results of the anodal tDCS group. **(A)** Mean group change of PT (±SEM) **(B)** individual data with high visual demand **(C)** individual data with low visual demand.

Mean pre PT value was 47.8 ± 9.2% maximum stimulator output (MSO) in the session with high visual demand and 45.8 ± 10.2% MSO in the session with low visual demand, respectively. Pre and post tDCS PT values were subjected to an rmANOVA with the within-factors SESSION (high/low visual demand) and TIME (pre, post 1, post 2). A main effect for the factor SESSION was found (F_(1,15)_ = 7.74, η^2^ = 0.34, p = 0.014), but not for the factor TIME (F_(2,30)_ = 1.03, p = 0.37) and no interaction (F_(2,30)_ = 0.12, p = 0.89). Mean group data as well as individual data are depicted in Fig 3.

### Cathodal tDCS

**Fig 4:**
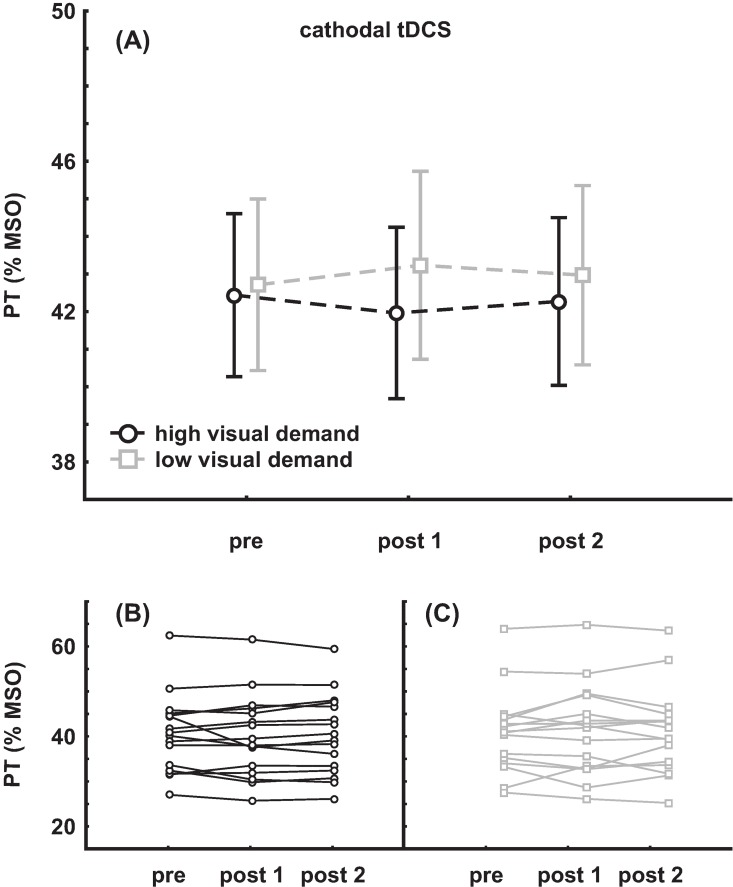
Results of the cathodal tDCS group. **(A)** Mean group change of PT (±SEM) **(B)** individual data with high visual demand **(C)** individual data with low visual demand.

Mean pre PT value was 42.4 ± 8.7% MSO in the session with high visual demand and 42.7 ± 9.1% MSO in the session with low visual demand, respectively. Pre and post tDCS PT values were subjected to an rmANOVA with the within-factors SESSION (high/low visual demand) and TIME (pre, post 1, post 2). No main effect was found (SESSION: F_(1,15)_ = 1.94, p = 0.18; TIME: F_(2,30)_ = 0.001, p = 0.99) and no interaction (F_(2,30)_ = 1.16, p = 0.33). Mean group data as well as individual data are depicted in Fig 4.

### External Factors

To evaluate whether the individual differences in response to tDCS and visual demand (see Fig 3B and 3C and Fig 4B and 4C) can be explained by external factors such as age, gender, baseline PT, slope of baseline PT or order of the sessions, additional analyses with respect to these factors were carried out.

The individual response to tDCS was calculated by subtracting the baseline PT value from the post 1 PT value, resulting in a delta tDCS PT value. Delta tDCS PT values > 0 indicate a numerical increase of PT, and delta tDCS PT values < 0 indicate a numerical decrease of PT following tDCS. Please notice that the variation between session 1 and session 2 took place after the post 1 PT measurement, so that the modulatory conditions for the two delta tDCS PT values were identical.

The individual response to visual demand was calculated by subtracting the post 1 PT value from the post 2 PT value, resulting in a delta visual demand PT value. Delta visual demand PT values > 0 indicate a numerical increase of PT, and delta visual demand PT values < 0 indicate a numerical decrease of PT following the visual demand period.

#### Age

No correlation between age of the subjects and baseline PT (mean session 1 and 2) was observed (r_s_ = 0.19, p = 0.30). Concerning the delta PT values, data were analyzed for the anodal and cathodal group as well as for the two sessions separately. For both groups, no correlation was found in any condition neither for response to tDCS (anodal group: r_s_ = -0.12, p = 0.67 high visual demand session; r_s_ = -0.02, p = 0.95 low visual demand session; cathodal group: r_s_ = 0.11, p = 0.68 high visual demand session; r_s_ = 0.24, p = 0.38 low visual demand session) nor for response to visual demand (anodal group: r_s_ = 0.18, p = 0.51 high visual demand; r_s_ = 0.50, p = 0.05 low visual demand; cathodal group: r_s_ = 0.41, p = 0.12 high visual demand; r_s_ = -0.26, p = 0.33 low visual demand). There is a trend to a significant correlation between age and increased PTs following low visual demand in the anodal group. However, all delta visual demand PT values were within the intra-individual baseline PT variation of about 3.9%MSO within sessions, with one exception showing a delta visual demand PT value of 6.6%MSO.

#### Gender

A two-sample t-test revealed lower baseline PTs (mean session 1 and 2) in females compared to males (t = 2.11, p = 0.04). Concerning the delta PT values, data were analyzed for the anodal and cathodal group as well as for the two sessions separately. There was no difference in response to tDCS between females and males in any condition (anodal group: t = 0.94, p = 0.36 high visual demand session; t = 0.18, p = 0.86 low visual demand session; cathodal group: t = 0.78, p = 0.45 high visual demand session; t = 1.06, p = 0.31 low visual demand session). Likewise, no difference in response to visual demand between females and males were observed (anodal group: t = 0.48, p = 0.64 high visual demand; t = 1.78, p = 0.10 low visual demand; cathodal group: t = 0.91, p = 0.38 high visual demand; t = 0.42, p = 0.68 low visual demand).

#### Baseline PT

Data were analyzed for the anodal and cathodal group as well as for the two sessions separately. For both groups, no correlation between baseline PT and delta tDCS PT value was found in any condition (anodal group: r_s_ = 0.45, p = 0.08 high visual demand session; r_s_ = -0.08, p = 0.78 low visual demand session; cathodal group: r_s_ = 0.15, p = 0.57 high visual demand session; r_s_ = 0.32, p = 0.23 low visual demand session). Likewise, no correlation between baseline PT and delta visual demand PT value was found in any condition (anodal group: r_s_ = -0.15, p = 0.59 high visual demand; r_s_ = 0.44, p = 0.09 low visual demand; cathodal group: r_s_ = 0.003, p = 0.99 high visual demand; r_s_ = -0.29, p = 0.28 low visual demand).

#### Slope of baseline PT

Recently, we suggested that the slope of the baseline PT might influence cTBS effects on visual cortex excitability [29]. Therefore, we evaluated whether there is a correlation between the slope of baseline PT and response to tDCS or visual demand as well. Data were analyzed for the anodal and cathodal group as well as for the two sessions separately. For both groups, no correlation was found in any condition neither for response to tDCS (anodal group: r_s_ = -0.17, p = 0.52 high visual demand session; r_s_ = -0.15, p = 0.58 low visual demand session; cathodal group: r_s_ = -0.32, p = 0.22 high visual demand session; r_s_ = 0.17, p = 0.53 low visual demand session) nor for response to visual demand (anodal group: r_s_ = 0.19, p = 0.47 high visual demand; r_s_ = -0.27, p = 0.31 low visual demand; cathodal group: r_s_ = -0.39, p = 0.14 high visual deman; r_s_ = -0.38, p = 0.15 low visual demand).

#### Order of sessions

A two-sample t-test revealed no difference in baseline PT between the first and second session (t = 0.86, p = 0.39). Since order of sessions (high/low visual demand) was counterbalanced across subjects, the significant interaction in the omnibus ANOVA showing lower baseline PTs in the session with low visual demand for the anodal group (Table 1), is not based on a systematic order effect, but rather at random.

Concerning the delta tDCS PT values, data were analyzed for the anodal and cathodal group as well as for the two sessions separately. There was no difference in response to tDCS between the first and the second session (anodal group: t = 1.47, p = 0.16; cathodal group: t = 0.76, p = 0.46).

### Reliability of individual response to tDCS

**Fig 5:**
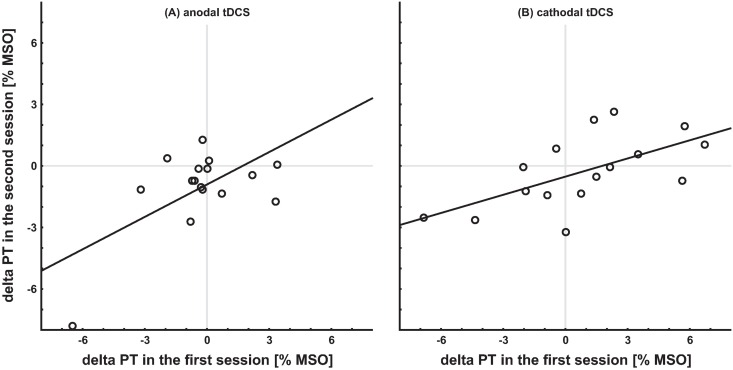
Correlation between the delta tDCS PT values of the two sessions. **(A)** anodal tDCS group: no significant correlation; r_s_ = 0.21, p = 0.44 **(B)** cathodal tDCS group: significant positive correlation; r_s_ = 0.63, p = 0.008.

Since we applied tDCS twice to each subject, we evaluated whether the individual response to tDCS (as indicated by the delta tDCS PT value) was reliable at any rate. Data were analyzed for the anodal and cathodal groups separately. No correlation between the delta tDCS PT values of the two sessions was observed for the anodal group (r_s_ = 0.25, p = 0.35, Fig 5A), but there was a significant positive correlation for the cathodal group (r_s_ = 0.63, p = 0.009, Fig 5B), indicating that at least for cathodal tDCS the individual response to tDCS might be reliable.

## Discussion

The aim of the study was to investigate the effects of anodal and cathodal tDCS on visual cortex excitability in dependency of whether there is low or high visual demand following stimulation. Based on the results of former studies, we hypothesized: (a) anodal tDCS will decrease PTs and cathodal tDCS will increase PT [16, 17]; (b) if subjects are exposed to low visual demand following tDCS, the modulatory effect will last for at least 10minutes [16]; and (c) if subjects are exposed to high visual demand following tDCS, the modulatory effect will be modified, i.e., there will be a decrease in PT independent from tDCS polarity [23]. However, our results did not confirm any of these hypotheses since we observed no modulation of PT neither directly following tDCS nor following the visual demand periods. Since we discovered high inter-individual variability in the response to tDCS, we tried to identify external factors which may account for these differences. Unfortunately, none of our parameters investigated seems to be responsible for the differences or could predict the direction of tDCS effects. Furthermore, the change of PT was very small in most cases and might rather indicate common intra- individual variations in PT than a modulatory effect of tDCS, since subjects’ responses to tDCS varied between the two identical tDCS applications. Only for cathodal tDCS there was an intra-subject reliability in response to tDCS.

In recent years, many researchers applied tDCS to the visual cortex, investigating a variety of parameters. In the first studies decreased PTs following anodal tDCS and increased PTs following cathodal tDCS were observed, for both stationary [16] as well as moving PTs [17]. Similarly, tDCS was reported to modulate static and dynamic contrast sensitivities [19], the amplitude of VEPs [30] and VEP-related oscillatory activities [31]. Some of these observations (e.g., [18, 30, 32] confirm the canonical assumption “anodal excitatory, cathodal inhibitory” (AeCi- effect, [33]) derived from the first study in the motor system [6], which has been successfully replicated in quite a few studies (e.g., [34–36]). However, in many experiments a wide range of tDCS- effects in the visual cortex was observed, rejecting the AeCi- assumption. For instance, whereas cathodal tDCS indeed impaired contrast sensitivity, anodal tDCS had no effect [19]. Likewise, cathodal tDCS decreased the beta and gamma power of VEPs to elementary visual stimuli, but with anodal tDCS only a non- significant trend towards an increase was observed [31]. However, it was shown that the amplitude of VEPs can be modified by tDCS only using an OZ-CZ electrode montage [31]. It was suggested that the stimulation efficacy of tDCS over the visual cortex depends on current flow direction. In the present study, the electrode on the visual cortex was placed over the individual phosphene “hot-spot”, the coil position resulting in the clearest phosphene perception. Thus, it is conceivable that at least in some subjects rather O1 or O2 than OZ was targeted by tDCS. Indeed, this could have led to the non-findings presented here, since neither an O1-O2 electrode montage nor an OZ-left mastoid montage led to significant effects of tDCS on VEP amplitudes [31]. With respect to the electrode size, we would not expect a big difference in regional current flow between OZ-CZ and O1/O2-CZ montages, but a modeling approach comparing the two constellations would be informative.

Recently, in a meta-analysis it was shown that the AeCi-effect is rare in cognitive domains [33], and a systematic review on tDCS studies declared tDCS-effects to be little-to-no reliable beyond MEP amplitude modulation [24]. Indeed, a change of PT does not really belong to a cognitive domain, and in the review of Horvath et al. [24] no study on PTs was included. Nevertheless, our results fit well to those publications for two reasons. Firstly, the AeCi-effect could not be confirmed by our study. Secondly, our results are in contrast to at least one former study [16] showing the (AeCi-) expected polarity-specific effects of tDCS on stationary PT. Recently, it was criticized that there are five issues never being discussed in tDCS studies [37]. For instance, possible sources of inconsistency in tDCS experiments are intra-subject reliability and inter-subject variability. Up to now, there is no study investigating the intra-subject reliability regarding tDCS effects on the visual cortex. As our subjects were stimulated twice with exactly the same parameters, a reliability of the effect was seen for cathodal tDCS only. Anyway, in spite of cathodal reliability, the direction of the modulatory “effect” was inconsistent, showing high inter-subject variability. In a recent study using perfusion fMRI recordings of cerebral blood flow it was shown that individual subjects showed wide variability in their responses to cTBS, although the stimulation led to weak selective increases under the coil in cerebral blood flow measurements across the group [38]. The observed interindividual variability in these increases was related to changes in functional connectivity of the relevant intrinsic networks. Although the frontal cortex has been stimulated in their study, and they used cTBS instead of tDCS, their results correspond to our findings of large interindividual responses to brain stimulation.

Evaluating whether the individual differences in response to tDCS can be explained by external factors, we found no parameter predicting the individual tDCS effect. Neither age, gender, baseline PT, slope of baseline PT nor order of the sessions had an influence on the direction of the tDCS “effect”.

Furthermore, assuming that tDCS has no effect on PT, we would expect a modulation of visual cortex excitability at least in dependency of high or low visual demand. It was shown that light deprivation modulates PT [39], as well as being exposed to different luminance contrasts [40] or reading [23]. All these kinds of low or high visual demand periods were shown to change PT, but with our visual demand periods we failed to observe a systematic change of PT. Thus, the question arises of the main differences between the present observation and the above mentioned studies. First, let us consider PT measurement procedure. In our study the method of constant stimuli was used, applying 49 TMS pulses at 7 different stimulator output intensities in steps of 3% MSO [20] with intensities randomly intermixed to avoid hysteresis effects. A sigmoidal fit subsequently generates the individual PT at the reversal point of the logistic function [41]. Thus, in our study PT is defined as the TMS intensity generating a phosphene with a probability of 0.5. Usually, PT is evaluated by applying pulses with increasing intensity until a phosphene is perceived. Then, intensity is decreased in steps of about 5% until an intensity without any perception, followed by an increase in steps of 1–2% until again a phosphene is perceived (e.g., [42, 43] or perceived in 3 of 5 cases (e.g., [16, 17, 23]), with the latter version resulting in a phosphene probability of 0.6 at PT. Although some researchers determine PT by the use of adaptive paradigms (e.g., [40]), there is high variability in PT measurements between and within subjects, since, for instance, in that case a range of 6 to 35 pulses per PT measurement has been applied. However, these differences in PT determination methods are unlikely to cause the differences under consideration. Another methodological difference is the maintenance of coil position. In most of the studies measuring PT several times in the same subject the phosphene hotspot was marked on the subject’s scalp [16, 17, 42] or a swimming cap [12, 39]. In some studies, a chinrest was used and the coil was fastened with a clamp [40] or the position of the hotspot was measured using a measuring tape [23]. In our study, the exact location of the hotspot and the coil position is maintained by the use of a neuronavigation system, ensuring a good reliability of the stimulated cortex area within as well as between sessions. Another important parameter is the intraindividual variability in PT within and between sessions. In the present study, individual PT varies with a maximum of 3.9%MSO between the two baseline measurements in the same session and 6.5%MSO between sessions. Most of the tDCS “effects” observed here were below these values, rather indicating a normal variation within subjects than a modulatory effect. Unfortunately, in most of the former studies mean group differences were reported only. Although a baseline PT is measured at least twice in many studies, intraindividual variation have not been reported [16, 17, 23]. In some studies, baseline PT correlations between sessions were evaluated [42–44] and always a high correlation of about 0.61 [43], 0.7 [44] or 0.93 [42] was observed. However, having a closer look to the figures in these publications, intraindividual variation in PT is quite large: a variation between the two PT measurements in the same subject of up to 13% ([42] Fig 1A), 16% ([43] Fig 1) or even 20%MSO can be seen ([44] Fig 3). In combination with a usually small sample size of 9 [16, 17] or 12 subjects [23], large intraindividual PT differences can probably pretend modulatory effects of tDCS. In our study, anodal as well as cathodal tDCS was applied twice to 16 subjects, respectively, resulting in 32 anodal and 32 cathodal tDCS sessions. All of the methodological differences between our study and the other publications described do, in our view, rather improve the reliability and validity of PT with respect to the former studies. However, a direct comparison of the studies suffer by differences in other parameters like different stimulators, coils, units of PTs or normalization of PT values to baseline. As mentioned above, there are some important issues never discussed in tDCS studies [37], such as inter-subject variability and intra-subject reliability. Taking these issues into account, we clearly failed to observe any systematic effect of tDCS on visual cortex excitability. In our view, future studies should report individual data together with mean group data in order to contribute to characterizing brain stimulation effects and its replicability. Additionally, raw data should be included, in particular if effects are reported on the basis of normalized data.

## Supporting Information

S1 Table Raw data. Demographic data and dependent values of 32 subjects.
(XLSX)
